# Transforming cancer treatment: integrating patient-derived organoids and CRISPR screening for precision medicine

**DOI:** 10.3389/fphar.2025.1563198

**Published:** 2025-03-25

**Authors:** Ziyi Zhu, Jiayang Shen, Paul Chi-Lui Ho, Ya Hu, Zhaowu Ma, Lingzhi Wang

**Affiliations:** ^1^ The First Affiliated Hospital of Yangtze University, Yangtze University, Jingzhou, Hubei, China; ^2^ School of Basic Medicine, Yangtze University, Health Science Center, Yangtze University, Jingzhou, Hubei, China; ^3^ School of Pharmacy, Monash University Malaysia, Subang Jaya, Malaysia; ^4^ Department of Pharmacology, Yong Loo Lin School of Medicine, National University of Singapore, Singapore, Singapore; ^5^ Cancer Science Institute of Singapore, National University of Singapore, Singapore, Singapore; ^6^ NUS Centre for Cancer Research (N2CR), National University of Singapore, Singapore, Singapore

**Keywords:** patient-derived organoids, CRISPR screening, precision medicine, cancer treatment, immunotherapy

## Abstract

The persistently high mortality rates associated with cancer underscore the imperative need for innovative, efficacious, and safer therapeutic agents, as well as a more nuanced understanding of tumor biology. Patient-derived organoids (PDOs) have emerged as innovative preclinical models with significant translational potential, capable of accurately recapitulating the structural, functional, and heterogeneous characteristics of primary tumors. When integrated with cutting-edge genomic tools such as CRISPR, PDOs provide a powerful platform for identifying cancer driver genes and novel therapeutic targets. This comprehensive review delves into recent advancements in CRISPR-mediated functional screens leveraging PDOs across diverse cancer types, highlighting their pivotal role in high-throughput functional genomics and tumor microenvironment (TME) modeling. Furthermore, this review highlights the synergistic potential of integrating PDOs with CRISPR screens in cancer immunotherapy, focusing on uncovering immune evasion mechanisms and improving the efficacy of immunotherapeutic approaches. Together, these cutting-edge technologies offer significant promise for advancing precision oncology.

## 1 Introduction

Cancer is a leading cause of incidence and mortality worldwide (Bray et al., 2024). Despite significant advancements in treatment modalities, including immunotherapy, targeted therapy, and precision medicine (Zeng et al., 2023), challenges such as therapy resistance, tumor heterogeneity, and metastatic progression continue to limit therapeutic efficacy and patient outcomes (Rulten et al., 2023). For instance, resistance to immune checkpoint inhibitors affects up to 60%–70% of patients, while targeted therapies often fail due to the emergence of secondary mutations. This emphasizes the urgent need for *in vitro* models that closely mimick tumors *in vivo* and developing more effective therapeutic strategies to deepen our understanding of cancer biology and to support the development of precise and personalized treatment strategies. In this context, patient-derived organoids (PDOs) have emerged as a transformative platform, recapitulating tumor complexity and microenvironmental interactions to bridge the gap between traditional models and clinical translation.

In recent years, PDOs have emerged as a pivotal tool in cancer research and therapy. PDOs are three-dimensional (3D) cell culture systems derived from patient tumor tissue that retain the genetic variability and phenotypic diversity characteristic of the primary tumor (Weeber et al., 2017; Verstegen et al., 2025). Unlike traditional two-dimensional (2D) cell cultures and patient-derived xenografts (PDXs), PDOs are able to mimic the tumor microenvironment (TME), enabling the study of interactions between cancer cells and their surroundings and the assessment of therapeutic response in a patient-specific manner (Shi et al., 2024). Moreover, PDOs provide a new *in vitro* model for disease modeling, drug screening and precision medicine in personalized treatment of diseases, while also uncovering therapeutic targets and mechanisms of drug resistance to enhance treatment strategies (Wang Q. et al., 2022).

CRISPR technology has transformed cancer research through precise genetic editing and functional screening, enabling the systematic exploration the roles of driver genes in cancer progression and treatment response (Xue et al., 2020). By creating knockout or activation libraries, it identifies genetic vulnerabilities in cancer cells and reveals interactions between genetic changes and drug responses, supporting targeted therapy development. Moreover, CRISPR screening can provide insight into the interplay between various genetic alterations and therapeutic response, thereby informing the development of targeted therapies (Shi et al., 2023).

The integration of CRISPR screening with PDOs offers a physiologically relevant platform to dissect genetic dependencies within native TME, accelerating the translation of functional genomics insights into precision oncology strategies and personalized treatment. PDOs enable the evaluation of drug sensitivity, while CRISPR-based functional screens identify novel therapeutic targets, paving the way for more effective and individualized therapies (Mundekkad and Cho, 2022). Additionally, this combined approach enhances the understanding of immune evasion mechanisms in tumors, potentially improving immunotherapy strategies. By overcoming drug resistance and refining treatment options, this integration holds promise for better patient outcomes and more precise cancer therapies (Yang et al., 2022).

This review highlights the latest advancements in PDOs, CRISPR screening in cancer research, and their combined potential applications, including studies on oncogenesis, tumor progression and metastasis. It underscores the urgent need for innovative approaches in precision oncology and cancer immunotherapy, emphasizing the transformative role of these technologies in bridging the gap between laboratory research and clinical practice. By advancing personalized cancer treatments, particularly immunotherapies, this integration offers significant promise for improving outcomes for cancer patients.

## 2 PDOs and their applications

### 2.1 Cultivation techniques and classification of PDOs

PDOs allow the culture of 3D organ-like structures from patient tissues that retain the genetic and phenotypic characteristics of the primary tumor, providing a more accurate model for studying tumor biology and detecting response to therapy, and representing a major advance in cancer research (Yan et al., 2018). PDOs can be categorized on the basis of their origin, such as tumor-derived organoids from cancerous tissue for tumor-derived organoids, and from healthy tissue for normal tissue-derived organoids. In addition, PDOs can be classified based on their specific applications in research and clinical settings, including drug testing, disease modeling, and personalized medicine (Banerjee and Senapati, 2024).

The establishment of PDOs has revolutionized the field of cancer research by providing a more accurate representation of the tumor microenvironment (TME) than traditional 2D cell cultures. This enhanced fidelity allows for a better understanding of tumor biology, drug response, and the development of targeted therapies (Huang et al., 2024). The establishment of PDOs involves the isolation of cells from a tumor biopsy and their cultivation in a special medium that supports their growth and differentiation. This process not only preserves tumor heterogeneity, but also facilitates the study of the TME, which is critical for understanding cancer progression and treatment resistance (Tong et al., 2024). PDOs have been effectively established from numerous cancer types, such as pancreatic, colorectal, and breast cancers. Moreover, these PDOs have demonstrated their utility in facilitating drug susceptibility assessments and in pinpointing prospective therapeutic targets (Xia et al., 2019), highlighting their potential in the realm of personalized medicine. The culture of PDOs requires meticulous optimization of culture conditions to ensure their viability and function. Various factors, such as the extracellular matrix, the selection of growth factors, and the composition of the culture medium, play a crucial role in the successful establishment and maintenance of PDOs. For example, the use of Matrigel as a scaffold has been shown to enhance structural integrity and growth of organoids, while specific combinations of growth factors can promote differentiation and more closely mimic the *in vivo* environment (Hirt et al., 2022). Recent studies have also explored the use of microfluidic systems to create organoid models on a chip, allowing for better control of the microenvironment and facilitating high-throughput drug screening (Qu et al., 2021).

### 2.2 Applications of PDOs

The applications of PDOs are immense and impactful, especially in the fields of cancer research and precision medicine. PDOs have helped elucidate the mechanisms of cancer progression and response to therapy, providing insights into tumor heterogeneity and the role of the TME in therapeutic outcomes (Li et al., 2024). PDOs retain the structural integrity and cellular heterogeneity inherent to the primary tumor, including cancer stem cells, differentiated cells, and stromal components, and are essential for studying the mechanisms of tumor behavior and evaluating drug efficacy (Shi et al., 2024). The ability of PDOs to be built up from a wide range of cancer types, including breast, colorectal, pancreatic, gastric, hepatocellular, and other cancers, makes them it a universal model for cancer research (Baskar et al., 2022). Furthermore, to study the complex interactions that occur within tumors, by co-culturing PDOs with stromal or immune cells, the key to how the microenvironment promotes tumor growth, metastasis, and therapeutic resistance has been revealed. For example, studies using PDOs have shown that cancer-associated fibroblasts enhance tumor cell survival and proliferation while also modulating the immune response (Wang Q. et al., 2022; Bulcaen et al., 2024).

PDOs are used to conduct drug screening in addition to studying disease mechanisms, including hereditary, infectious, and malignant diseases (Wang et al., 2023; Han et al., 2024). PDOs allow for the identification of drug sensitivities and resistances against individual patients, and can serve as valuable platforms for drug screening to evaluate therapeutic agents on patient-specific tumor models for efficacy and toxicity (Tang et al., 2022). Studies have shown that drug response in PDOs is strongly correlated with patient prognosis, making them a powerful tool for personalized medicine (Wang et al., 2022b). For example, PDOs have been used to identify effective drug combinations for the treatment of colorectal cancer (CRC), highlighting their potential for optimizing treatment strategies (Huang et al., 2024). Recent studies have also highlighted the ability of PDOs to predict patient response to chemotherapy and targeted therapies, highlighting their potential as a platform for the development of individualized treatment regimens (Zhou Z. et al., 2021; Yu et al., 2022). To conclude, the establishment of patient-derived organoids marks a key advancement in cancer research and treatment, providing a powerful platform for studying tumor biology, optimizing therapeutic strategies, and personalizing patient care. As research continues to evolve, PDOs will play an increasingly integral role in the future of precision oncology.

## 3 CRISPR screening technology and its applications

### 3.1 Mechanism and advantages of the CRISPR-Cas9 system

The CRISPR-Cas9 system has revolutionized the field of genetic engineering and molecular biology, providing an efficient and precise method for gene editing (Pacesa et al., 2024). The technology originated in the adaptive immune system of prokaryotes and utilizes short RNA sequences to guide the Cas9 nuclease to specific DNA sequences for targeted modifications (Cong et al., 2013; Mali et al., 2013). CRISPRCas9-medied genome editing to achieve activation/silencing of the target sequence of the target gene is completed in accordance with the three steps of guided RNA target recognition of the target gene sequence, Cas9 nuclease upstream of the target sequence to break the double-strandedness of the target gene, and ligating or repairing the double-strandedness of the break (Asmamaw and Zawdie, 2021). Two of the key components are Cas9 endonuclease and guide RNA (gRNA). gRNA is designed to be complementary to a specific DNA sequence in the target gene, allowing Cas9 proteins to introduce double-stranded breaks at precise locations in the genome, which can be rapidly customized for different target genes. This mechanism enables a variety of gene modifications, including gene knockouts, knock-ins, and transcriptional regulation, which can be used for therapeutic purposes and functional genomics studies (Chen et al., 2019; Li C. et al., 2023).

The versatility of CRISPR-Cas9 has led to its widespread use in different fields, especially in cancer research, where it has been used to explore gene function, build tumor models and identify therapeutic targets (Wang S. W. et al., 2022). Recent advances have introduced derived technologies such as CRISPR interference (CRISPRi) and CRISPR activation (CRISPRa). In addition, CRISPR/Cas9 can target multiple genes at the same time (multiplexing), which is particularly beneficial for the study of complex traits and diseases involving multiple genetic factors (Yu et al., 2022). For example, through the development of the STING-seq method, which integrates genetic association studies, gene editing, and single-cell sequencing, CRISPR screening has been used to establish the link between genetic variation and human health, and to explore genetic predispositions, which provide a basis for understanding the genetic basis of biological processes (Morris et al., 2023). In another study, we integrated multi-omics and CRISPR screening technology to screen chondrocytes for genome-wide knockout and analyze the mechanism of genetic predisposition for height, which highlights the central role of CRISPR screening in analyzing genetic predisposition and exploring key genetic factors, and provides new perspectives and tools for biomedical research (Richard et al., 2025). The low-cost and high-throughput capabilities of CRISPR-Cas9 allow for a wide range of applications in various fields, including agriculture, biotechnology, and medicine (Lu et al., 2021). Despite its advantages, challenges such as off-target effects and delivery methods remain key areas of research to improve the safety and efficacy of CRISPR-based interventions (Zhang et al., 2021).

### 3.2 Applications of CRISPR screening

Functional CRISPR screens provide valuable insights into the mechanisms of cancer progression and treatment response by enabling high-throughput identification of key genes contributing to tumorigenesis, drug resistance, and metastasis (Chandrasekaran et al., 2022), an approach that reveals novel oncogenic drivers and tumor suppressor genes (Sharma and Giri, 2024). These screens utilize the CRISPR system to introduce targeted gene disruptions throughout the genome, enabling the systematic study of gene function in cancer cells. A significant advantage of CRISPR screens is their ability to reveal both loss-of-function and gain-of-function mutations, providing a comprehensive understanding of the role of genes in tumorigenesis (Tang, 2024; Wang and Doudna, 2023).

CRISPR screening techniques can be categorized into three main types: CRISPR knockout (CRISPR KO), CRISPRi and CRISPRa. Each method manipulates gene expression in different ways, facilitating the exploration of gene function in different biological environments. For example, CRISPR KO screens are widely used to identify essential genes in cancer cells, helping to reveal pathways leading to tumorigenesis and drug resistance (Zhao et al., 2022).

In the field of immunotherapy, CRISPR screening plays a pivotal role in uncovering critical factors within the tumor microenvironment (TME), enabling the identification of valuable biomarkers to enhance the effectiveness of existing treatments (Cao et al., 2024). For example, CRISPR screening conducted in both comparative 2D cultures and xenografts derived from the same cell line demonstrated the dual role of MEN1 in regulating tumor-microenvironment interactions, offering significant insights for advancing cancer immunotherapy (Su et al., 2024). Furthermore, CRISPR screening techniques have been employed to formulate innovative therapeutic approaches, such as gene therapy and precision medicine. This is achieved by enhancing the genetic underpinnings of diseases and facilitating the creation of targeted treatment strategies (Li Y. R. et al., 2023). A genome-wide CRISPR screening was conducted on CD8-positive T cells to uncover the regulatory factors influencing tumor infiltration in a murine model of triple-negative breast cancer. The findings of this research highlighted established immunotherapeutic targets, including Pdcd1/PD-1 and Hacvr2/Tim-3, while also uncovering novel targets, including Dhx37, that had not been previously reported (Dong et al., 2019). As the field advances, the integration of CRISPR technology with new organoid models is expected to unleash cancer research, greatly improving the treatment outcome of cancer patients.

## 4 Integration of CRISPR screens with PDOs in cancer research

### 4.1 Advantages of CRISPR-PDO integration

**FIGURE 1:**
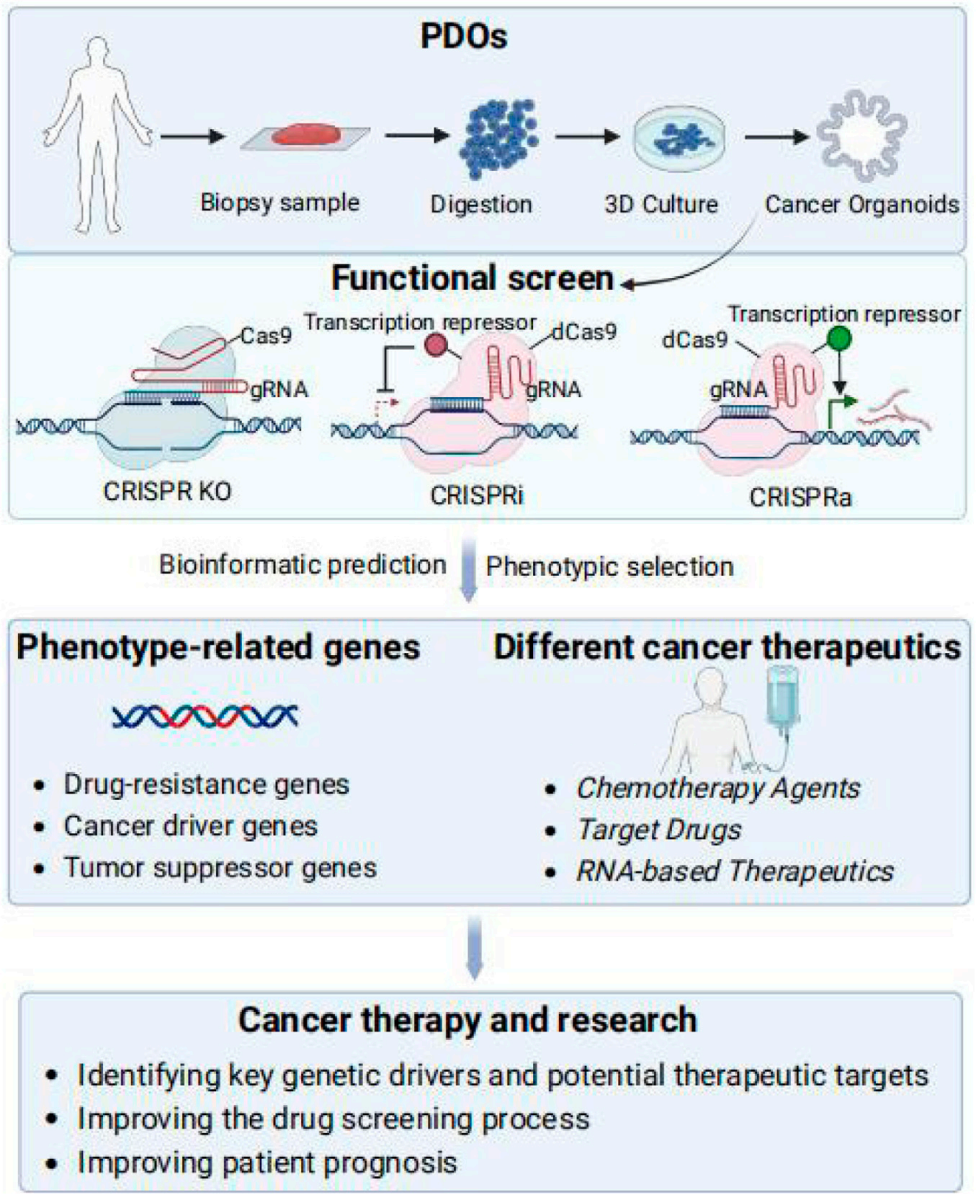
Integration of PDOs and functional CRISPR screens. Functional screening in organoids can be realized via CRISPR-Cas screening technologies, including CRISPR KO, CRISPRa, and CRISPRi. After screening for phenotypes, bioinformatics prediction and screening are utilized to identify phenotype-associated genes, drug resistance genes and TSGs as potential therapeutic targets to reverse drug resistance or in combination with drugs provides new methods for cancer treatment and research, including identifying key genetic drivers of cancer and potential therapeutic targets, improving the drug screening process, and enhancing patient prognosis.

The integration of PDOs with CRISPR screening offers a transformative approach to cancer research and personalized medicine. The fidelity of PDOs allows for the assessment of drug response and the exploration of therapeutic strategies tailored to individual patients. When combined with CRISPR technology to enable precise gene editing and functional genomics, the use of bioinformatics prediction and screening to identify phenotype-associated genes provides new approaches for reversing drug resistance or combining drugs for cancer treatment and research, with important implications for cancer treatment and research, including identifying key genetic drivers and potential therapeutic targets for cancer, improving the drug screening process, and improving patient prognosis (Figure 1).

A key advantage of utilizing CRISPR screens in PDOs is that it helps to identify phenotype-related genes such as drug resistance genes, cancer driver genes and tumor suppressor genes, unearth potential therapeutic targets, and provide direction for the development of targeted cancer therapies (Ravi et al., 2023). For instance, CRISPR-cas9-mediated gene editing of cancer organoids has been used to validate cancer driver genes and to understand the mechanisms of drug resistance potential targets for therapeutic intervention (Ray and Mukherjee, 2021; Wang H. et al., 2021). Furthermore, the drug screening process can be improved to enhance screening efficiency and accuracy and accelerate anti-cancer drug development. An unbiased genome-wide CRISPR loss-of-function screen in HCC organoids revealing WEE1’s vulnerability to oxaliplatin and enhanced therapeutic response to the WEE1 inhibitor adavosertib combination therapy (Jia et al., 2025). By penetrating research into cancer mechanisms and optimizing drug screening, it is expected to find more effective treatment options and improve patient outcomes.

The integration of CRISPR screens with PDOs aids in studying gene-environment interactions in the TME by coculturing organoids with immune cells, revealing how genetic changes in cancer cells affect their microenvironment interactions, crucial for understanding immune evasion and cancer immunotherapy strategies (Takeda, 2021). Moreover, combining CRISPR screens with single-cell RNA sequencing in PDOs has shed light on cancer cell heterogeneity and responses to genetic changes, helping identify subpopulations linked to therapeutic resistance or relapse (Wang et al., 2022d), such as novel targets in breast cancer organoids post-CRISPR knockout (Vishnubalaji and Alajez, 2023).

Together, the integration of CRISPR screens with PDOs represents a powerful approach for dissecting the genetic and molecular underpinnings of cancer. This combined strategy not only enhances our understanding of tumor biology but also has accelerated the discovery of new therapeutic targets and the development of personalized treatment strategies. The subsequent sections delve deeper into specific case studies and the latest advancements in this rapidly evolving field.

### 4.2 Applications in cancer research

**FIGURE 2:**
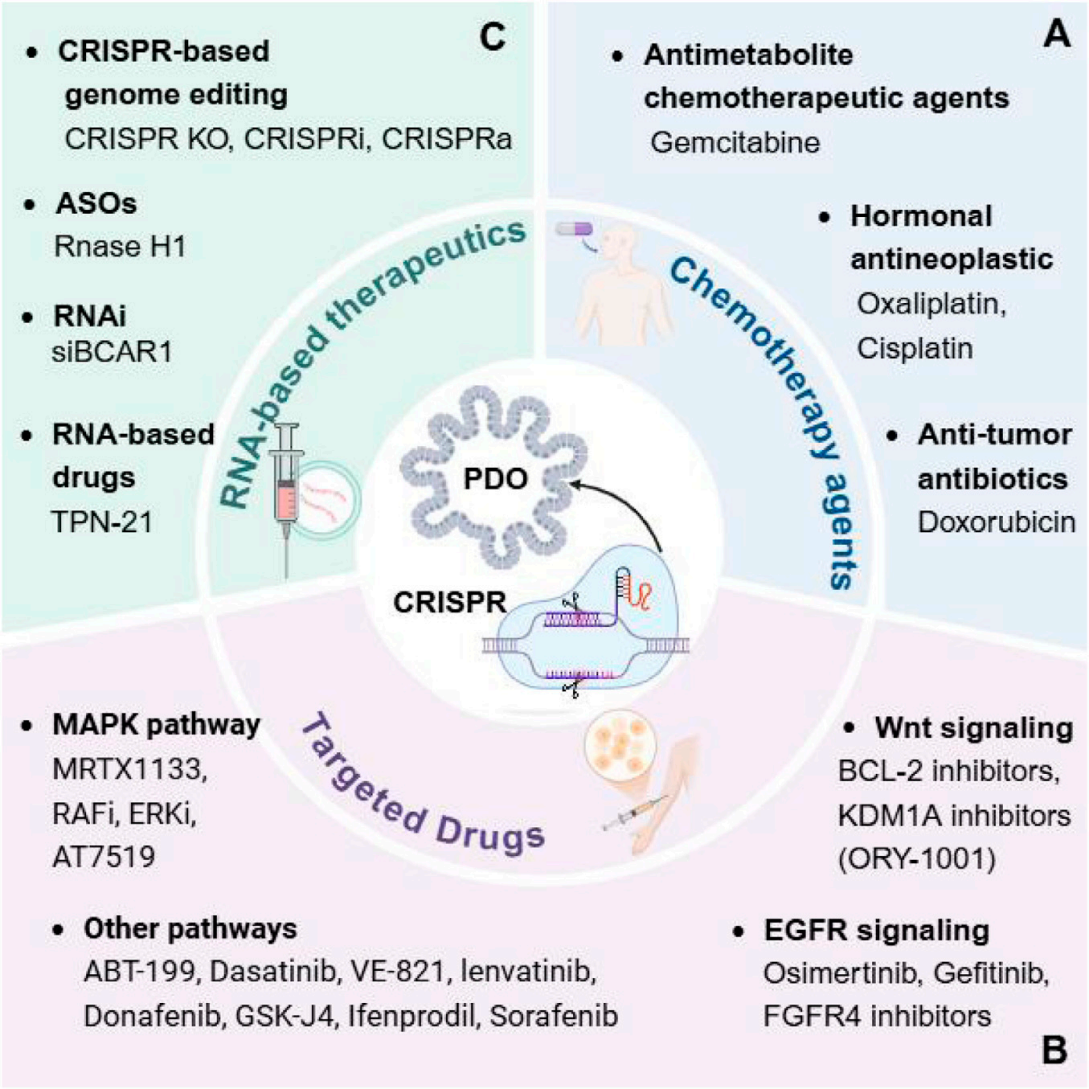
Using CRISPR screens in cancer organoids to identify different cancer therapeutics. CRISPR screens for drugs in different cancer PDOs, such as chemotherapy agents **(A)**, targeted drugs **(B)**, and RNA-based therapeutics **(C)**, which in combination with potential therapeutic targets in the screened tumors can reverse resistance or combine them to further improve drug efficacy.

The absence of an *in vitro* tumor model that accurately recapitulates the heterogeneity of human cancers significantly impedes our comprehension of cancer pathogenesis and the assessment of treatment efficacy and toxicity (Marusyk et al., 2020). 3D organoid culture models have demonstrated considerable potential in the representation of human cancers (Kuo and Curtis, 2018; Muthuswamy, 2018; Crespo et al., 2017; Di Modugno et al., 2019). The integration of CRISPR technology aids in the identification of targetable mutations and dysfunctional signaling pathways that are essential for the survival and growth of tumor cells (Stratton et al., 2009). In the following sections, we will focus on these molecular aberrations to identify genes that can inhibit tumor growth and metastasis or reverse drug resistance (Figure 2; Table 1). This approach aims to enhance the efficacy of cancer therapies, reduce drug toxicity, and minimize side effect to heathy cells.

**TABLE 1:** Identifying different cancer therapeutics in cancer organoid models using CRISPR screening.

Cancer types	Organoid models	Drug types	Drugs	Functions and mechanisms	References
Colorectal Cancer	OXL-resistant cell lines and organoids	Chemotherapy agents	Oxaliplatin	MIEF2 may serve as a specific biomarker of OXL responsiveness and a potential target for the development of therapeutic approaches to improve chemotherapy efficacy	Xie et al. (2022)
Bladder Cancer	PDOs	Chemotherapy agents	Cisplatin	Intracellular drug transport: NPEPPS affects cisplatin sensitivity by regulating drug concentrations	Jones et al. (2024)
Pancreatic Cancer	PDAC organoids	Chemotherapy agents	Gemcitabine	DNA Replication Stress Resistance: APOBEC3C and APOBEC3D roles identified	Ubhi et al. (2024)
Osteosarcoma	Human organoids	Chemotherapy agents	Doxorubicin	AKT phosphorylation: PRKDC recruited and bound GDE2 to enhance the stability of GNAS, activated AKT phosphorylation and conferred resistance to DOX.	Zhang et al. (2024)
Hepatocellular Carcinoma	HCC PDOs	Chemotherapy agents	Metformin	RAC1 activation, Cell survival: Metformin promotes DOCK1 phosphorylation, leading to resistance	Feng et al. (2022)
Gastric Cancer	Tumorsphere and organoid	Targeted drug	BLC2 inhibitors	Wnt, YAP signaling: NF2 and RASA1 deficiency amplifies these pathways in cancer stem cells	Kwon et al. (2023)
Gastric Cancer	GC PDOs	Targeted drug	KDM1A inhibitors	Wnt signaling, Cell cycle: KDM1A inhibition leads to inhibition of Wnt signaling and G1 cell cycle arrest	Mircetic et al. (2023)
Lung Cancer	Organoids	Targeted drug	Osimertinib, Gefitinib	Hippo pathway: Regulates resistance to EGFR inhibitors	Pfeifer et al. (2024)
Breast Cancer	PDXs and PDOs	Targeted drug	FGFR4 inhibitors	β-catenin/TCF4 signaling pathway, SLC7A11/FPN1 Axis:Involved in glutathione synthesis and Fe2+ efflux, linked to ferroptosis induced by FGFR4 inhibition	Zou et al. (2022)
Breast Cancer	PDOs *in vitro* and cell-derived xenograft or PDXs *in vivo*	Targeted drug	CDK12 inhibitor	PI3K/AKT signaling pathway: Inhibition of CDK12 suppresses this pathway, impacting resistance to lapatinib	Li et al. (2021)
Pancreatic Cancer	PDAC PDXs	Targeted drug	MRTX1133(KRAS G12D inhibitor)	Mechanotransduction, YAP/TAZ expression: ITGB1 influences these pathways to impact KRAS inhibitor efficacy	Kumarasamy et al. (2024)
Pancreatic Cancer	Cell line, organoid, and rat models of PDAC	Targeted drug	RAFi, ERKi	RAF-MEK-ERK cascade: Low-dose vertical inhibition strategy for KRAS mutant PDAC	Ozkan-Dagliyan et al. (2020)
Pancreatic Cancer	3D organoids	Targeted drug	AT7519(CDK1, CDK2, CDK7, CDK9 inhibitor)	CDK network: Hyperactivation linked to mt KRas dependency	Kazi et al. (2021)
Breast Cancer	PDO and PDX models	Targeted drug	ABT-199 (BLC2 inhibitor)	BCL2 pathway: Targeted in combination with CDK4/6 inhibitors for ER + breast cancer	Whittle et al. (2020)
Pancreatic Cancer	PDAC cell lines and organoids	Targeted drug	CDK4/6i, ERKi	CDK4/6, ERK-MAPK, PI3K-AKT-mTOR: Combination therapies targeting these pathways	Goodwin et al. (2023)
Pancreatic Cancer	Human PDAC organoid biobank	Targeted drug	Dasatinib, VE-821	Kinase inhibitor sensitivity: ARID1A mutations associated with sensitivity to dasatinib and VE-821	Hirt et al. (2022)
Hepatocellular Carcinoma	Patient-derived primary organoid	Targeted drug	Lenvatinib	Lenvatinib resistance: LAPTM5 could promote intrinsic macroautophagic/autophagic flux by facilitating autolysosome formation	Pan et al. (2023)
Hepatocellular Carcinoma	Animal and PDO models	Targeted drug	Lenvatinib	Mitophagy, antioxidant pathways: LINC01607 regulates these pathways affecting Lenvatinib resistance	Zhang et al. (2023)
Hepatocellular Carcinoma	PDX and PDO models	Targeted drug	Donafenib, GSK-J4	Ferroptosis:Induced by upregulation of HMOX1 and increased intracellular Fe2+ level	Zheng et al. (2023)
Hepatocellular Carcinoma	HCC PDOs and human tumor xenograft models	Targeted drug	Ifenprodil, Sorafenib	Unfolded protein response, WNT signaling, Stemness: Targets of ifenprodil and sorafenib combination	Xu et al. (2021)
Ovarian Carcinoma	RB1-deficient patient HGSC organoids	Targeted drug	Carboplatinum	Cell cycle regulation: CK2 inhibition affects RB family cell cycle regulator p130	Bulanova et al. (2024)
Colorectal Cancer	CRC PDOs	Targeted drug	HDAC inhibitors	H3K9 acetylation and H3K9 dimethylation:Targets of HDAC and EHMT1/2 inhibition	Bamberg et al. (2022)
Colorectal Cancer	Wild-type and APC mutant human intestinal organoids	RNA-based therapeutics	-	TGF-β pathway: Resistance to TGF-β-mediated growth restriction	Ringel et al. (2020)
Colorectal Cancer	Pre-malignant organoids with APC^−/−^;KRASG12D mutations	RNA-based therapeutics	-	TGF-β pathway: Used as a paradigm for screening in organoids	Michels et al. (2020)
Colorectal Cancer	PDOs	RNA-based therapeutics	-	Transplantation of *in vitro* CRISPR-edited cells enables high-throughput and CRISPR-based single-guide rna screening in organoid transplantation to validate cancer cells including colorectal cancer at all stages of its development and treatment	Ray and Mukherjee (2021)
Colorectal Cancer	Primary CRC PDOs	RNA-based therapeutics	-	m6A-dependent oncogenic translation, Wnt signaling: Driven by YTHDF1 and RUVBL1/2	Chen et al. (2024b)
Colorectal Cancer	CRC PDOs	RNA-based therapeutics	-	IPO11 knockout decreased colony formation of CRC cell lines and decreased proliferation of patient-derived CRC organoids	Mis et al. (2020)
Colon Cancer	Colon cancer cells and 3D organoids	RNA-based therapeutics	-	ER stress and ROS production: Linked to stemness and drug resistance in colon cancer	Zhao et al. (2023)
Colorectal Cancer	PDO models	RNA-based therapeutics	-	EMT pathway: ANKRD42 regulates CRC distant metastasis	Liu et al. (2024)
Cholangiocarcinoma	Gene-mutant organoids	RNA-based therapeutics	-	PI3K pathway, Immune microenvironment, Inflammatory responses: Cul3 mutation alters immune microenvironment; other mutations affect various pathways	Feng et al. (2024)
Gastric Cancer	Mouse gastric epithelial organoids	RNA-based therapeutics	-	Wnt signaling: Alk, Bclaf3, Prkra regulate Wnt-driven stem cell-dependent epithelial renewal	Murakami et al. (2021)
Pancreatic Cancer	Tumor organoid cultures from colorectal carcinoma patients	RNA-based therapeutics	-	Wnt signaling: Circuit identified as a vulnerability in RNF43-mutant tumors	Denecke et al. (2023)
Breast Cancer	PDOs	RNA-based therapeutics	-	Overexpression of RNaseH1, a ribosomal endonuclease that specifically degrades the r-loop, rescued the reduced clonogenicity caused by TOP1 deletion, suggesting that this vulnerability is driven by aberrant r-loop accumulation	Lin et al. (2023)
Pancreatic Cancer	PDAC organoids	RNA-based therapeutics	-	SRC inhibitor-mediated inhibition of p130Cas phosphorylation impairs MYC transcription via a DOCK1-RAC1-β-catenin-dependent mechanism	Waters et al. (2021)
Pancreatic Cancer	PDO and PDX models	RNA-based therapeutics	-	Targeting aberrantly expressed oncogenic miRNAs and precisely delivering them to tumor cells with the help of nanocomplexes	Gilles et al. (2018)

#### 4.2.1 Chemotherapy agents

The identification of tumor targets to reverse drug resistance is critical in enhancing the efficacy of chemotherapy. Chemotherapeutic agents, including antimetabolites like 5-fluorouracil and anthracyclines, often face challenges due to the development of resistance mechanisms within cancer cells (Feng et al., 2023). By utilizing CRISPR screening in PDOs, genes genes implicated in drug resistance can be systematically knocked out, thus revealing potential targets for therapeutic intervention (Figure 2A).

For instance, the effectiveness of oxaliplatin (OXL), which serves as the primary chemotherapeutic drug for CRC, is frequently hampered by the emergence of drug resistance. In CRC PDOs, CRISPR/Cas9 screening technology has identified MIEF2 as a critical gene associated with resistance. Notably, the diminished expression of MIEF2 correlates with decreased mitochondrial stability and a suppression of apoptosis, thereby influencing the sensitivity of CRC to OXL treatment (Xie et al., 2022). In bladder cancer PDOs, multi-omics analysis and CRISPR screening revealed NPEPPS as a driver of cisplatin resistance, and depletion of NPEPPS was able to re-sensitize drug-resistant cells to cisplatin, whereas overexpression of NPEPPS generated resistance by regulating intracellular drug concentration (Jones et al., 2024). Gemcitabine is the main antimetabolite chemotherapeutic agent for pancreatic ductal adenocarcinoma (PDAC), but many tumors are resistant to it. In PDAC PDOs, a CRISPR-Cas9 screen revealed cytidine deaminases APOBEC3C and APOBEC3D as key genes responsive to gemcitabine that enhance cellular resistance to replication stress by promoting DNA replication fork restart and repair, providing a rational target for improved gemcitabine-based PDAC therapy (Ubhi et al., 2024). Anti-tumor antibiotics such as adriamycin (DOX) are key agents in chemotherapy for osteosarcoma, but the problem of drug resistance severely limits its efficacy. CRISPR screening identified PRKDC as a key determinant of sensitivity to DOX in osteosarcoma, which induces chemoresistance by recruiting GDE2 to stabilize GNAS and activate the AKT signaling pathway. Therefore, combining the PRKDC inhibitor AZD7648 with DOX significantly inhibited osteosarcoma growth in organoids, providing a new targeting strategy to improve the efficacy of osteosarcoma chemotherapy (Zhang et al., 2024).

The ability to model these interactions in PDOs allows for a more nuanced understanding of how tumors adapt to therapeutic pressures, paving the way for the development of combination therapies that can effectively overcome drug resistance.

#### 4.2.2 Targeted drugs

Unlike conventional chemotherapeutic agents that indiscriminately affect both cancerous and normal dividing cells, targeted therapies are designed to interfere with specific molecular targets that are involved in the growth, progression, and spread of cancer (Stratton et al., 2009). Cancer cells achieve tumor growth and metastatic dissemination through dysregulation of various signaling pathways, including maintenance of proliferative signaling, evasion of growth inhibitory factors, resistance to cell death, achievement of replicative immortality, induction of angiogenesis, and activation of invasion and metastasis (Hanahan and Weinberg, 2011). The dysregulation of various signaling pathways often underlies these hallmark traits, thereby presenting logical targets for therapeutic intervention. Therefore, the need to improve the efficacy of drugs targeting these signaling pathways is critical (Figure 2B).

One of the most critical pathways associated with cancer is the Wnt/β-catenin signaling pathway, which plays a key role in cell proliferation and differentiation (Clevers and Nusse, 2012). Using CRISPR screen in PDOs to identify resistance genes targeting these pathways may inhibit cancer cell proliferation and induce apoptosis. By CRISPR screening has identified the critical roles of Nf2 and Rasa1 as metastasis suppressor genes in cancer stem cells (CSCs) to regulate invasive features and metastatic ability through Wnt and YAP signaling pathways (Kwon et al., 2023). Furthermore, the result of CRISPR screen in GC PDOs showed that KDM1A inhibitor (ORY-1001) deregulated NDRG1 inhibition, which in turn inhibited Wnt signaling and cell cycle (Mircetic et al., 2023). These findings allow for a more nuanced understanding of how tumors adapt to therapeutic pressures, paving the way for the development of combination therapies that can effectively overcome resistance.

Similarly, dysregulated EGFR signaling, often due to receptor overexpression or mutation, enhances tumorigenesis by promoting uncontrolled cell division, impeding programmed cell death, and facilitating invasion and metastasis (Vaquero et al., 2022). A study suggests that HER2 amplification is associated with resistance to anti-EGFR therapy (Bertotti et al., 2015). For example, in breast cancer organoids, FGFR4 and CDK12 were identified by CRISPR/Cas9 screening as key factors in anti-HER2 treatment resistance in HER2-positive breast cancers. FGFR4 inhibition enhanced the sensitivity of drug-resistant tumors to HER2 treatment, whereas inhibition of CDK12 restored sensitivity to HER2 tyrosine kinase inhibitors (TKIs) (Zou et al., 2022; Li et al., 2021). These findings provide new therapeutic targets to overcome drug resistance in HER2-positive breast cancer.

The RAS/RAF/MEK/ERK signaling pathway, frequently referred to as the MAPK pathway, constitutes a crucial cascade that orchestrates processes such as cellular proliferation, survival, and differentiation. Among the various genetic modifications observed in human malignancies, mutations in the KRAS gene are recognized as some of the most prevalent alterations (Takeda et al., 2019). Recent advances have led to the development of covalent inhibitors targeting specific KRAS mutations, e.g., studies have revealed the potential of MRTX1133 targeting KRAS G12D in PDAC therapy, and a CRISPR screen identified ITGB1 as a key target for enhancing the response to MRTX1133 therapy by regulating mechanotransduction signaling and YAP/TAZ expression (Kumarasamy et al., 2024).

In addition to these three common pathways, there are some drugs that target other pathways. CDK4/6 inhibitors significantly prolong tumor response in patients with metastatic estrogen receptor-positive (ER+) breast cancer, but recurrence is almost inevitable (Spring et al., 2020). A CRISPR/Cas9 screen showed that ABT-199 is a potent and selective BCL2 inhibitor that in combination with CDK4/6 inhibitors has potential for the treatment of ER + breast cancer (Whittle et al., 2020). VEGF is a potent angiogenic factor that is inhibited by drugs targeting the VEGF receptor such as signaling tyrosine kinase inhibitors (e.g., sunitinib and sorafenib) (Lee et al., 2015). However, drug resistance limits clinical efficacy. A CRISPR-Cas9 screen identified that the combination of the NMDAR antagonist Ifenprodil with sorafenib significantly increased antitumor activity in HCC PDOs (Xu et al., 2021). PARP inhibitors exploit synthetic lethality by targeting the DNA repair pathway. A CRISPR-Cas9 screen revealed a significant increase in the antitumor activity of the NMDAR antagonist ifenprodil in RB1-deficient HGSC and TNBC-like organs, CK2 inhibitors were able to enhance sensitivity to PARP inhibitors, providing a novel strategy to overcome therapeutic resistance in these cancers (Bulanova et al., 2024). The study also identified countless new targets and pathways with tumor therapeutic potential. These include targeting the TME, modulating the cancer epigenome, inhibiting the proteasome and interfering with cancer metabolism. Targeting specific molecular aberrations, such as by CRISPR-Cas9 screening, we found that knockdown of EHMT1/2 significantly enhanced the anti-proliferative effect of HDAC inhibitors in CRC, suggesting that EHMT1/2 is a potential epigenetic therapeutic target (Bamberg et al., 2022).

On the basis of the PDOs model, the use of CRISPR screening not only aids in the identification of novel therapeutic targets but also informs the design of combination therapies that can preemptively address potential resistance mechanisms, ultimately enhancing the efficacy of targeted treatments in clinical settings. By integrating these methodologies, researchers can better navigate the complexities of tumor biology and improve therapeutic strategies for patients facing drug-resistant cancers.

#### 4.2.3 RNA-based therapeutics

Compared to conventional protein-targeted and DNA-based medicines, RNA-based therapeutics hold great promise due to their distinct physicochemical and physiological properties (Crooke et al., 2018). RNA-based therapeutics represent a rapidly evolving frontier in the treatment of various diseases, particularly in oncology, cardiovascular diseases, and genetic disorders. These therapies utilize RNA molecules to modulate gene expression and protein synthesis, providing a targeted approach to disease management (Figure 2C).

ASOs are short, single-stranded oligonucleotides that can pair with specific RNA complementary bases to reduce, restore or modify protein expression by can alter RNA (Crooke et al., 2021). One of the degradation mechanisms mediated by RNaseH1 is also known as enzymatic RNA degradation (Liang et al., 2017). A genome-wide CRISPR knockdown screen revealed TOP1 as a potential target for synthetic lethality in MYC-dysregulated breast cancer cells. Overexpression of RNaseH1 reversed the decrease in clone-forming ability induced by TOP1 deletion, highlighting its importance in regulating aberrant r-loop accumulation and DNA repair. It may also affect gene expression and DNA repair processes by regulating RNA-DNA hybrids, which may have an impact on cancer cell survival and proliferation (Lin et al., 2023).

RNAi induces double-stranded RNA degradation of specific RNA targets, providing an intrinsic defense mechanism against invading viruses and transcription factors (Kim and Rossi, 2007). SiRNAs can induce RNAi in mammalian cells (Castanotto and Rossi, 2009), thereby establishing this targeted gene silencing mechanism as a robust experimental approach for functional genomics research. BCAR1 was identified as a potential therapeutic target through the use of siRNA screening and CRISPR-Cas9 genomic screening in pancreatic cancer-like organs. Inhibition of BCAR1 enhanced the sensitivity of pancreatic cancer cells to ERK inhibitors, while the combination of SRC inhibitors and microtubule protein inhibitors synergistically reduced MYC protein levels and effectively inhibited the growth of KRAS-mutant pancreatic cancers (Waters et al., 2021).

The prokaryote-derived CRISPR-Cas system has been widely used in mammalian cells and organisms for precise genome sequence editing, which is an efficient genome editing (Doudna and Charpentier, 2014). SgRNAs play the role of guides in the CRISPR-Cas9 system, and by designing specific sgRNA sequences, they are able to guide the Cas9 proteins to specific sites in target DNA sequences, enabling the cleavage and editing of specific locations in the genome (Michels et al., 2020). One study systematically demonstrates the utility of sgRNA-based screening platforms in elucidating TGF-β signaling components that drive colorectal cancer (CRC) progression, highlighting their potential as therapeutic targets (Ringel et al., 2020).

While CRISPR-based therapeutics hold the potential to precisely target virtually any genomic locus, their clinical translation remains contingent on the development of delivery systems capable of ensuring cell-specific uptake while circumventing unintended immune activation (Madigan et al., 2023). Recent advancements in nanotechnology have significantly enhanced the delivery systems for RNA-based drugs, improving their stability and bioavailability (Ren et al., 2012). The therapeutic potential of a nanomedicine, TPN-21, was first demonstrated to reduce tumor cell growth and survival in individual patients with PDO avatars. By utilizing PDO as a rapid screen for TPN-21, RNA-based oligonucleotides packaged in a TPN targeting a specific PDAC tumor receptor demonstrated that TPN-21 strongly inhibits PDO growth even in the presence of gemcitabine failure (Gilles et al., 2018).

However, the most significant obstacle preventing the widespread usage of RNA-based approaches is the difficulty of efficiently delivering such drugs to target organs and tissues apart from the liver. Most delivery methods for these RNA-based therapeutics can be categorized as the addition of targeting moieties, the encapsulation of RNAs into lipid-based nanoparticles, and direct delivery into the target organ without extensive modification. In addition, off-target binding (Kamola et al., 2015), sequence-induced toxicity, and oversaturation of the endogenous RNA processing pathway affect the effectiveness of RNA-based approaches (Grimm et al., 2006). Despite the inherent limitations of current RNA-based therapeutic platforms, ongoing mechanistic studies, including exogenous RNA delivery strategies and small molecule-mediated RNA targeting approaches, promise to enable the systematic development of next-generation RNA-targeted drug therapeutics with improved clinical translational potential.

## 5 Therapeutic implications of PDOs and CRISPR screens in cancer immunotherapy

Cancer immunotherapy has emerged as a revolutionary approach in oncology, harnessing the body’s immune system to fight malignancies. The promise of immunotherapy is immense, as evidenced by the remarkable advances in immune checkpoint inhibitors and CAR-T cell therapy, which have shown significant efficacy in a variety of cancers, including melanoma and hematologic malignancies (Ni et al., 2023). Despite these advancements, significant challenges persist. Many patients exhibit primary or acquired resistance to these treatments, limiting their effectiveness (Bockamp et al., 2020). In addition, an immunosuppressive barrier often exists in the TME, preventing immune cell infiltration and activity (Bader et al., 2020). Tumor heterogeneity further complicates treatment, as different tumor types and even subtypes within a single tumor may respond differently to immunotherapeutic agents. In addition, the risk of immune-related adverse events poses a significant problem that requires a careful balance between efficacy and safety (Meier et al., 2024). Addressing these challenges is critical for the successful incorporation of immunotherapy into standard cancer care, highlighting the need for innovative strategies to improve outcomes.

The integration of PDOs with CRISPR technology represents a groundbreaking innovation in cancer immunotherapy research. This approach not only deepens our understanding of tumor biology but also enables personalized medicine by allowing the screening of patient-specific responses to a broad range of immunotherapeutic agents. Additionally, the capability to model the TME in PDOs offers critical insights into mechanisms of immune evasion, paving the way for the development of more effective immunotherapies that can overcome these barriers.

**FIGURE 3:**
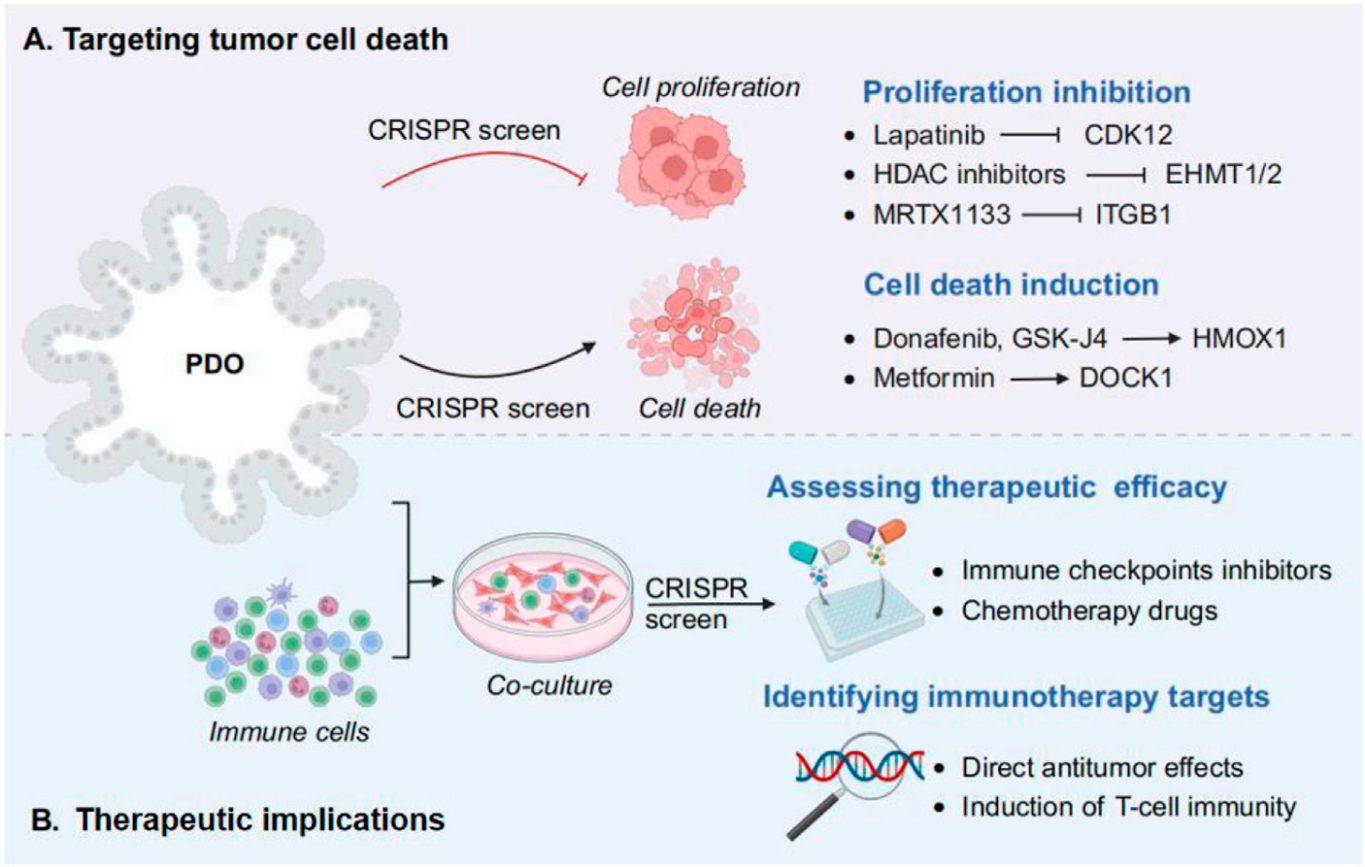
Using CRISPR screens in PDOs for cancer treatment strategies. **(A)** Targeting tumor cell death. The role of phenotype related genes and drugs in tumor cell death mechanism, including inhibition of cell proliferation (e.g., CDK12, EHMT1/2, and ITGB1), and induction of cell death (e.g., HMOX1 and DOCK1). **(B)** Therapeutic implications. By incorporating immune cells into PDO culture, followed by CRISPR screening to assess the efficacy of drug therapy and identify the immunotherapy targets.

The integration of CRISPR-Cas9 technology with PDOs screen for phenotype-related genes that can target tumor cell death in cancer immunotherapy, including inhibition of cell proliferation and induction of cell death (Figure 3A). Importin-11 (IPO11) was identified as an essential factor for β-catenin–mediated transcription in APC-mutant CRC cells by a genome-wide CRISPR screen. IPO11 knockdown reduced colony formation in CRC cell lines and reduced proliferation of patient-derived CRC-like organs, revealing that IPO11 promotes β-catenin nuclear import that cancer therapy by reducing cell proliferation (Mis et al., 2020). Similarly, in HCC, the strategic application of CRISPR-Cas9 screening has significantly advanced the identification of synergistic drug combinations that enhance cell death induction. For instance, the combination of donafenib with GSK-J4 has been shown to promote ferroptosis in liver cancer models, a process driven by the synergistic upregulation of HMOX1 expression alongside increased intracellular iron levels (Zheng et al., 2023). These developments signify a paradigm shift towards personalized and precision oncology, wherein genetic insights are leveraged to design tailored therapeutic strategies that effectively inhibit unchecked cancer cell proliferation, revolutionizing the landscape of cancer treatment.

The amalgamation of PDOs with CRISPR-Cas9 technology has also opened new avenues by assessing therapeutic efficacy and identifying immunotherapy targets in cancer therapy (Figure 3B). Compared with traditional 2D cultures or animal models, PDOs provide a more accurate representation of the TME, making them invaluable for preclinical testing of immunotherapeutic agents. By incorporating immune cells into PDO cultures, researchers can better mimic the interactions between cancer cells and the immune system, thereby enhancing the predictive power of these models for immunotherapy responses (Grönholm et al., 2021). For example, studies have demonstrated that systematically dissect PDA intrinsic mechanisms of immune evasion by CRISPR screening in PDA organiods, and identify Vps4b and Rnf31 as essential factors required for escaping CD8 T cell killing (Frey et al., 2022). Therefore, PDOs can be used to evaluate the efficacy of immune-related targets and various immunotherapeutic approaches, thereby shedding light on the mechanisms underlying immune evasion and resistance (Xiang et al., 2024).

CRISPR-Cas9 technology further enhances the utility of PDOs by enabling precise genetic modifications. This allows for the systematic investigation of gene functions and the identification of novel therapeutic targets. In recent years, the gene editing technology of CRISPR-Cas9 has been used in clinical trials of human immunotherapy (Sun et al., 2022). Immunotherapy based on gene editing technology mainly modifies T cells to recognize tumor antigens and increase anti-tumor activity (Osborn et al., 2016). By knocking out specific genes in PDOs, researchers can study the effects of these genetic alterations on tumor-immune interactions and identify potential vulnerabilities that can be targeted by immunotherapies (Sun et al., 2023). CheckCell-2 (NCT05566223), a CRISPR/Cas9-based CISH gene therapy, knockdown of CISH in CD8 (+) T cells improves the efficacy of cancer immunotherapy. CD70 effectively regulates T cells and promotes immunosuppression in the TME, a target for immunotherapy. CTX131 (NCT05795595) and CTX130 (NCT04438083), immunotherapies targeting allogeneic CD70 CAR T cells, enhance the efficacy of CAR T cell therapy for relapsed and refractory solid tumors (Dewulf et al., 2023; Feng et al., 2020). Moreover, the combination of PDOs and CRISPR-Cas9 technology has facilitated the development of personalized cancer immunotherapy approaches. By creating organoids from individual patients’ tumors and using CRISPR to introduce specific genetic modifications, researchers can tailor immunotherapeutic strategies to the unique genetic and immunological landscape of each patient’s cancer (LaFleur and Sharpe, 2022). This personalized approach not only improves the efficacy of immunotherapies but also reduces the risk of adverse effects by targeting the specific pathways involved in each patient’s tumor (Dao et al., 2022).

In summary, the integration of PDOs with CRISPR-Cas9 technology represents a powerful tool for advancing cancer immunotherapy. By providing a more accurate and personalized model of the TME, this approach facilitates the discovery of novel therapeutic targets, the assessment of immunotherapeutic agents, and the formulation of personalized treatment strategies. With advances in novel technologies, it offers significant potential to increase the effectiveness of cancer immunotherapy and bring us closer to the goal of precision oncology (Bonaventura et al., 2022).

## 6 Conclusion and future perspectives

In the rapidly evolving landscape of cancer research, the integration of PDOs and functional CRISPR screens has emerged as a transformative approach, offering unprecedented insights into tumor biology and therapeutic vulnerabilities. This review emphasizes the integration of PDOs and CRISPR screening in tumor therapy and their potential therapeutic applications. Additionally, we highlight common applications of both technologies in tumor progression to reverse drug resistance or perform combination therapy by targeting multiple signaling pathways associated with cell proliferation, apoptosis, and metastasis through screened potential targets. Importantly, combined PDOs and CRISPR screening can explore new potential therapeutic targets for cancer as well as new targeted drugs for more precise individualized medicine. The combined use of PDOs and CRISPR screening may provide important hints for understanding the biological functions of cancer and provide new clinical value for individualized cancer medicine.

While combining organoid and CRISPR screening has shown attractive breakthroughs and prospects, the field is yet to conquer certain challenges and limitations. Firstly, current techniques for PDOs culture are inherently uncontrollable and non-repeatable due to several non-standardized aspects of cancer tissue source and subsequent processing, media formulation, and animal-derived 3D matrices. For example, culture media containing multiple growth factors and nutrients because of the inclusion of some of these components as purified recombinant proteins may also be limited by poor solubility and insufficient long-term storage stability, resulting in diminished protein activity. Moreover, the use of animal-derived sera introduces heterologous components that may adversely affect embedded organ models and limit human-specific immunological studies, as well as the risk of animal-derived, bacterial, or viral infections (LeSavage et al., 2022). To solve this difficulty, standardized media formulations can be developed to reduce heterogeneity and non-replicability of components. Secondly, despite the ability of organoids to mimic many features of neural development, the lack of some important physiological processes, including vascularization and transmission of signal mediators remains a key issue. For example, brain organoids are prone to necrosis in the central part due to the lack of blood vessels, which affects their normal development and neuronal migration (Qian et al., 2019). The microfluidic chip can simulate dynamic perfusion, combining microfluidic technology with 3D-like blood vessels, supplemented with perfusion functions on the basis of having a vascular structure to fully simulate real blood perfusion (Bhatia and Ingber, 2014; Kistemaker et al., 2025; Birtele et al., 2024). Thirdly, potential gene targets or drug targets obtained through PDOs and CRISPR screening have clinical translation uncertainties. They may not have the expected therapeutic effect *in vivo*, and may even produce adverse reactions, increasing the risk and cost of clinical translation. Their effectiveness and safety in clinical treatment need further validation. The assessment of the safety of gene therapy can be briefly summarized in two aspects, assessment of genomic integrity (Pacesa et al., 2022; Jones et al., 2021) and identification of off-target editing activity (Cameron et al., 2023; Newton et al., 2019). Currently, development of new genetic screening techniques, such as CRISPR-StAR (Stochastic Activation by Recombination) technology can significantly improve the reliability and resolution of data, reducing the risks and costs of clinical translation (Uijttewaal et al., 2024).

Despite current challenges and limitations, combining PDOs and CRISPR screening with other technologies can further develop precision medicine and enhance individualized care. Artificial intelligence (AI)-driven drug discovery employs machine learning and deep neural networks to predict drug-target interactions and optimize molecular designs, overcoming cost and efficiency barriers of traditional methods (Li and Zhang, 2013; Hopkins, 2008; Krishnamoorthy et al., 2024; Lucas et al., 2024), while network pharmacology maps drug-polypharmacology networks to identify multi-target intervention strategies, particularly in oncology, by analyzing pathway interconnectivity and enabling mechanism-based therapeutic repositioning (Both et al., 2023; Reyna et al., 2022; Wang X. et al., 2021; Zhao et al., 2024; Zhou W. et al., 2021). Through the combination of these two technologies, off-target effects are systematically predicted at the preclinical stage and drug combinations are rationally designed to circumvent resistance mechanisms in tumor therapy (Mayer et al., 2024). AI-enhanced analysis of PDOs enables real-time therapy optimization (Co et al., 2024), while CRISPR-AI integration accelerates multi-target discovery through machine learning-prioritized gene editing (Jakavonytė-Staškuvienė and Mereckaitė-Kušleikė, 2023). Network pharmacology further empowers this framework by mapping drug-pathway polypharmacology, establishing a tripartite precision medicine platform in PDOs that synergizes clinical data mining, genetic target identification, and multi-target therapeutic design (Joshi et al., 2024). Therefore, AI and integrated network pharmacology in the fusion of PDOs and CRISPR screening techniques is expected to improve the accuracy and effectiveness of precision medicine. Moreover, CRISPR-engineered PDOs enable complex mutation modeling and scalable disease interrogation (Geurts and Clevers, 2023), while integration with single-cell multiomics, RNA-targeted editing, and immune-competent coculture systems provides unprecedented resolution in mapping tumor-immune dynamics (Ferreira da Silva et al., 2024; Chen Y. et al., 2024), synergistically advancing personalized therapeutic discovery.

In conclusion, integrating PDOs with functional CRISPR screening offers transformative potential for cancer research and therapy. These innovative approaches can expedite the identification of novel therapeutic targets, refine drug screening methodologies, and significantly improve patient outcomes. To unlock their full potential in the fight against cancer, ongoing interdisciplinary collaboration and technological advancements will be indispensable. Future research should focus on validating these targets in clinical trials, exploring combination therapies, or investigating their role in other cancer types to further translate these findings into effective treatments. By addressing these issues, we hope to bridge the gap between basic research and clinical applications, and ultimately contribute to the development of more precise and effective cancer therapies.

## Abbreviations

PDOs, patient-derived organoids; CRISPR, clustered regularly interspaced short palindromic repeats; 3D, three-dimensional; 2D, two-dimensional; PDXs, patient-derived xenografts; TME, tumor microenvironment; CRC, colorectal cancer; GC, gastric cancer; HCC, hepatocellular carcinoma; CRISPR KO, CRISPR knockout; CRISPRa, CRISPR activation; CRISPRi, CRISPR interference; gRNA, guide RNA; OXL, oxaliplatin; PDAC, pancreatic ductal adenocarcinoma; DOX, adriamycin; TGF-β, transforming growth factor-beta; TKIs, tyrosine kinase inhibitors; IPO11, Importin-11; TSG, tumor suppressor gene; StAR, Stochastic Activation by Recombination.
